# *Gnetum montanum* extract induces apoptosis by inhibiting the activation of AKT in SW480 human colon cancer cells

**DOI:** 10.1080/13880209.2022.2063340

**Published:** 2022-05-19

**Authors:** Xianglong Pan, Xiaotao Hou, Fan Zhang, Peiling Tang, Wanruo Wan, Zixia Su, Yeguo Yang, Wei Wei, Zhengcai Du, Jiagang Deng, Erwei Hao

**Affiliations:** a Guangxi Key Laboratory of Efficacy Study on Chinese Materia Medica, Guangxi University of Chinese Medicine, Nanning, People’s Republic of China; b Sino-Canada Joint Zebrafish Lab for Chinese Herbal Drug Screening, Guangxi University of Chinese Medicine, Nanning, People’s Republic of China; c Guangxi Collaborative Innovation Center for Research on Functional Ingredients of Agricultural Residues, Guangxi University of Chinese Medicine, Nanning, People’s Republic of China; d Guangxi Key Laboratory of TCM Formulas Theory and Transformation for Damp Diseases, Guangxi University of Chinese Medicine, Nanning, People’s Republic of China; e Department of Bioscience, Faculty of Applied Sciences, Tunku Abdul Rahman University College, Kuala Lumpur, Malaysia

**Keywords:** Yao medicine, flow cytometry, synergistic anticancer activity, cell cycle, AKT signalling pathway, chemical composition, zebrafish

## Abstract

**Context:**

*Gnetum montanum* Markgr. (Gnetaceae) is used to treat rheumatic arthralgia and bruises in the clinic.

**Objective:**

To exam the activity and mechanism of *G. montanum* extract (GME) against colon cancer cells SW480.

**Materials and methods:**

The anti-proliferative activity of GME (0–120 μg/mL) on SW480 cells was determined using MTS assay at 24, 48, and 72 h. The *in vitro* activity of GME (0–120 μg/mL) on SW480 cells was investigated using flow cytometry and western blotting analysis. The *in vivo* activity of GME was evaluated using xenograft tumour model of zebrafish and nude mice. The chemical composition of GME was detected by using HPLC–MS/MS.

**Results:**

The IC_50_ value SW480 cells viability by GME were 126.50, 78.25, and 50.77 μg/mL, respectively, for 24, 48, and 72 h. The experiments showed that apoptotic cells and G2/M phase cells increased from 20.81 to 61.53% (*p* < 0.01) and 25.76 to 34.93% with 120 μg/mL GME, respectively. GME also down-regulated the protein expression of P-AKT, P-GSK-3β, P-PDK1, P-c-Raf, caspase-3, and Bcl-2, and up-regulated the expression cleaved caspase-3, cleaved PARP, and Bax. *In vivo* study found that GME can significantly inhibit the growth and migration of SW480 cells in xenograft zebrafish. GME reduced the nude mice tumour weight to approximately 32.19% at 28 mg/kg/day and to 53.17% (*p* < 0.01) at 56 mg/kg/day. Forty-two compounds were identified from the GME.

**Discussion and conclusions:**

GME has a significant antitumor effect on colon cancer cells SW480, and it has the potential to be developed as an anticancer agent.

## Introduction

Colorectal cancer (CRC) is among the top three cancers with higher incident and mortality rate around the world. In 2020, the International Agency for Research on Cancer (IARC) documented that approximately 1.15 million of new CRC cases (which is about 10% of all types of cancers) and >570,000 of CRC-related deaths (which is about 9.4% of all cancer-related deaths) were reported worldwide (Jung et al. 2020; Sung et al. 2021). Colon cancer is a malignant tumour grows from the epithelium mucosal crypts of the large intestine (Iqbal and George 2017). Gene mutations (such as adenomatous polyposis (APC), deleted in colorectal cancer (DCC), K-Ras, p53, B-Raf proto-oncogene serine/threonine kinase (BRAF), mismatch repair gene) and microsatellite instability are the common factors that led to the development of colon cancer (Ahmed 2020; Benson et al. 2021). The common treatments of colon cancer include surgery, radiotherapy, chemotherapy, and molecular targeted therapy. Although the present diagnostic and therapeutic procedures have greatly been improved, the prognosis of colon cancer remains poor (Binefa et al. 2014; Li et al. 2021). The therapeutic drugs used to treat malignant tumours include chemo drugs, new technology drugs, and natural drugs. Research in exploring the novel natural compounds that can modulate apoptosis pathway of cancer cells for new drug development is intensively on-going. Various traditional plants with known medicinal properties are widely studied over the past decades (Hou et al. 2016; Aiello et al. 2019).

Apoptosis refers to the physiological death process of cells mediated by genes in accordance with their own pathway under a specific physiological or pathological condition to maintain the homeostasis (An et al. 2019; Ismail et al. 2019). According to Shariati and Meric-Bernstam (2019), AKT signalling pathway is closely related to cell apoptosis. AKT, also known as protein kinase B (PKB), is a serine/threonine kinase that mediates cell metabolism, proliferation, protein synthesis, survival and apoptosis (Liu et al. 2014; Manning and Toker 2017). It can be phosphorylated over 9000 proteins and it is an attractive therapeutic target in cancer (Mundi et al. 2016). Its kinase activity is positively mediated by the phosphorylation of the two key residues Thr^308^ and Ser^473^ (Liu et al. 2019). The activation of AKT signalling pathway can be governed by mainly phosphorylated AKT, glycogen synthase kinase-3β (GSK-3β), phosphoinositide dependent kinase-1 (PDK1) and Raf-1 proto-oncogene, serine/threonine kinase (c-Raf; Bamodu et al. 2020; Jiang et al. 2020). During cell cycle progression, AKT is phosphorylated and inhibits GSK-3β to prevent cyclin D1 degradation. Besides, PDK1, the 66-kD protein kinase phosphorylates AKT at Thr^308^. Phosphorylation of AKT negatively associated with the regulation of Raf-1 (Jun et al. 2012; Shorning et al. 2020). Numerous studies demonstrated that activation of AKT cascade often results in tumour aggressiveness and drug resistance in various types of human cancer (Chan et al. 2014; Neophytou et al. 2021).

*Gnetum montanum* Markgr. (Gnetaceae) is an evergreen vine indigenous to southern China and Southeast Asia (Wang et al. 2009). In traditional clinical practice of Yao medicine, the stems and rhizomes of this plant are used to treat rheumatic arthralgia and bruises (Qin et al. 2002; Ma et al. 2017). Besides, modern chemistry research shows that the plant contains abundant stilbenes (Zhai et al. 2016), sterols (Zhou et al. 1989), flavonoids (Xiang et al. 2002), alkaloids (Martin et al. 2011) and other bioactive phytochemicals. Furthermore, modern pharmacological research also proved that the plant exhibited various biological activities such as antitumor, antioxidation, anti-inflammatory, and antibacterial (Li et al. 2004; Shen et al. 2017). Despite the antitumor activity of *G. montanum* has been proven, the active compounds that contribute to the activity and its mechanism of action are not clear. In the previous pharmacodynamic study, we found that the stems and rhizomes of *G. montanum* exhibited significant inhibition on the proliferation of human colon cancer SW480 cells.

In this study, the chemical composition of *G. montanum* extract (GME) was initially identified using liquid chromatography–mass spectrometry (LC–MS). Then, the *in vitro* and *in vivo* inhibitory and apoptosis-inducing activities of GME on the growth of human colon cancer were determined. Besides, the mechanism of antitumor action of GME through AKT signalling pathway was also investigated. This research provides a fundamental scientific knowledge on the antitumor activity of *G. montanum* for future novel use and innovative development of GME.

## Materials and methods

### Cells, reagents and antibodies

The human colon cancer SW480 cells were obtained from the Cell Resource Centre, Institute of Life Sciences, Chinese Academy of Medical Science. L-15/Pen-Strep medium, foetal bovine serum (FBS), and 0.25% trypsin were purchased from Gibco (Grand Island, NY, USA). The CellTiter 96AQueous One Solution Cell Proliferation Assay (MTS) kit was obtained from Promega (Madison, WI, USA). The FITC Annexin V Apoptosis Detection Kit I and PI/R Nase Staining Buffer were purchased from BD Pharmingen (Franklin Lake, NJ, USA). 5-Fluorouracil (5-FU) was obtained from MedChemExpress (Franklin Lake, NJ, USA), whereas acetonitrile and ethanol (UPLC grade and analytical) were purchased from Fisher Scientific (Leicestershire, UK). The Pierce™ BCA Protein Assay Kit was purchased from Thermo Fisher (Rockford, IL, USA). The primary antibodies against caspase-3, cleaved caspase-3, PARP, cleaved PARP, Bcl-2, Bax, GAPDH and Phospho-AKT Pathway Antibody Sampler Kit were obtained from Cell Signalling Technology (Beverly, MA, USA).

### Animals and ethics

The AB strain of adult zebrafish was purchased from the China Zebrafish Resource Center (Wuhan, China). Zebrafish were maintained under standardized conditions at 28 °C in a 14 h light/10 h dark cycle (Subendran et al. 2021). Zebrafish were raised in the Guangxi Key Laboratory of Efficacy Study on Chinese Materia Medica (Nanning, China).

The 4–6 weeks old BALB/c strain of nude mice (male, 18.0 ± 2.0 g) was provided by the Hunan Silaike Jingda Laboratory Animal Co., Ltd. (Changsha, China). The mice were fed with food and water available *ad libitum* and were allowed to acclimatize for 1 week before the experiments. All experiments were conducted according to standard ethical guidelines and approved by the research ethical committee of Guangxi University of Chinese Medicine (approval number DW20200712-086).

### Preparation of plant extracts

The stems and rhizomes of *G. montanum* were collected in Jinxiu Yao Autonomous County, Guangxi Province, China in 2019. The species of the plant was identified by Prof. Wei Songji of School of Pharmacy, Guangxi University of Chinese Medicine. The plant is preserved in the Guangxi Key Laboratory of Efficacy Study on Chinese Materia Medica. The stems and rhizomes were processed to produce GME at the Guangxi Key Laboratory of Efficacy Study on Chinese Materia Medica to obtain GME. Briefly, 1 kg of dried stems and rhizomes were ground into powder using a heavy-duty blender, then soaked in 15 L of 95% ethanol. Extraction was carried out in a rotary shaker at a speed of 120 rpm for 24 h. Extraction was repeated for three times. The residues were separated through filtration. The three batches of filtrates were combined, then concentrated under reduced pressure. The extraction rate was 20% under this procedure. The extract was freeze-dried and stored at −20 °C until further usage.

### HPLC-Q-TOF-MS/MS analysis

Chemical constituents of GME were identified using a HPLC-Q-TOF-MS/MS system (put in brand). The separation of chemical components in GME was conducted in an ACQUITY HPLC BEH C18 column (2.1 mm × 100 mm, 1.7 μm, Waters Corporation, USA) using the mobile phase composed of 0.1% formic acid in water (A) and acetonitrile (B) at gradient elution programmed as follows: 0–40 min, 5–95% B; 40–42 min, 95% B; 42–43 min, 95–5% B; 43–48 min, 5% B. The flow rate was fixed at 0.4 mL/min. The temperature of column and autosampler were maintained at 40 and 4 °C, respectively. The injection volume was fixed at 3 μL. Mass spectrometric detection was conducted using a high-resolution AB SCIEX X500R quadrupole-time of flight (QTOF) mass spectrometer (HRMS) (Applied Biosystems SCIEX, USA) at a full scan mode from *m/z* 100 to 2000 under ESI mode operating in negative mode (Wei et al. 2021).

The MS and MS/MS data of the compounds were acquired in the information-dependent acquirement (IDA) technology mode. The optimized parameters for IDA were set as follows: ion source gas 1 (GS1) at 55 psi, ion source gas (GS2) at 55 psi, temperature at 600 °C and CAD gas at 7. For TOF MS: mass range of the components at *m/z* 100–2000, declustering potential (DP) at 80 V, collision energy (CE) at 35 V and CE spread at 0 V. For TOF MSMS: mass range of the fragments at *m/z* 100–2000, declustering potential (DP) at 80 V CE spread at 15 V, collision energy (CE) at 35 V and accumulation time at 0.05 s. Data acquisition and analysis were carried out using SCIEX OS software (Ver.1.3.1, AB SCIEX 153 Co.).

### Cell culture

The SW480 cells were grown in L-15/Pen-Strep medium supplemented with 10% FBS and incubated at 37 °C in a humidified atmosphere containing 5% CO_2_.

### MTS assay

The viability of SW480 cells against GME was assessed by MTS assay. The normal human colonic epithelial cell line NCM460 was used as the experiment control. Both cells were seeded in 96-well plate at a density of 3 × 10^3^ cells/well and allowed to adhere overnight. The cells were then treated with GME at concentration in the range of 10–120 μg/mL for 24, 48, and 72 h, respectively. Dimethylsulphoxide (DMSO) at a concentration of 0.1% was used as negative control. Next, 20 μL of MTS solution was added to each well at the end time points and incubated at 37 °C for 2 h. Absorbance at a wavelength of 490 nm was determined using a microplate reader. Cell viability rate (%) was calculated using a formula: (OD_treated group_ – OD_blank group_)/(OD_control group_ – OD_blank group_) × 100, where OD_treated group_, OD_control group_ and OD_blank group_ represent absorbance of SW480 cells. The half-maximal inhibitory concentration (IC_50_) was calculated by the GraphPad prism 8. Data were presented as the average of three independent experiments (Luo et al. 2019).

### DAPI staining

The SW480 cells were treated with GME at concentration in the range of 0–120 μg/mL for 48 h. DMSO at a concentration of 0.1% was used as negative control. Briefly, the cells were fixed with paraformaldehyde, then stained with 4′,6-diamidino-2-phenylindole (DAPI) for 10 min in the dark. After washing with cold PBS, the cells were observed under the Operetta CLS High-content analysis system (PerkinElmer, CA, USA) at DAPI pathway. Nine randomly selected areas of each group were photographed.

### Colony-forming assay

The SW480 cells were seeded in a six-well plate at a density of 400 cells per well. After 24 h, the cells were treated with 0.1% DMSO (negative control) and GME at different concentrations (10, 20, 40, 80, and 120 μg/mL), respectively, until interaction was observed between the cell colonies. The medium was then replaced with fresh medium containing 0.1% DMSO or GME at different concentrations every 4 days. The cultures were fixed with 4.0% paraformaldehyde and stained with crystal violet to determine colony formation. Typical images were captured and scanned.

### Migration assay

The inhibition of tumour cells migration by GME was performed by wound-healing assay. The SW480 cells were allowed to grow into full confluence in 6-well plates, then a vertical wound was created with a 200 μL micropipette tip. The cell debris was removed and fresh complete medium containing GME at concentrations of 40 and 80 μg/mL were added, respectively. The cells were incubated at 37 °C in the atmosphere of 5% CO_2_ for 24 h. Then, the cells were photographed using a microscope at three-time points (0, 24, and 48 h). The area of migration was measured and analyzed using Image J software. The experiments were conducted in triplicate.

### Flow cytometry analysis

The SW480 cells were exposed to different concentrations of GME (30, 60, and 120 μg/mL) and 5-FU (20 μM) at a fixed time (48 and 72 h) in six-well culture plate (2 × 10^5^ cells per well). For the apoptosis analysis, the cells and supernatant were harvested and washed in Dulbecco’s Phosphate Buffered Saline (DPBS) solution. Then, the cells were resuspended to a concentration of 1 × 10^6^ cells/mL in 1 × binding buffer. Next, the treated cells were stained with Annexin V-FITC and PI for 15 min at room temperature in dark before flow cytometry analysis. Approximately 50,000 cells were evaluated for each sample. For cell cycle analysis, the treated cells were incubated in propidium iodide (PI) according to the manufacturer protocol of CycleTEST PLUS DNA Reagent Kit (BD PharMingen, CA, USA). The percentages of cells in G0/G1, S, and G2/M phases were determined by flow cytometry. The experiment was performed in triplicate.

### Synergistic anticancer effects

To determine whether GME has a synergistic anticancer effect, mixture of 5-FU with different concentrations of GME (20, 40, and 80 μg/mL) were used to evaluate the proliferation inhibition on SW480 cells using the MTS assay as described earlier (Luo et al. 2019). Antitumor activity of the drug combinations was determined using Median Effect Equation, whereby Fa/Fu = (D/Dm)m. Compusyn software was used to calculate the Combination Index (CI). CI <1 indicates synergism, CI = 1 indicates summation effect and CI >1 indicates antagonism (Chou and Talalay 1984).

### Western blotting analysis

After treating SW480 cells with different concentrations of GME for 72 h, total protein of SW480 cells was extracted and determined using BCA Protein Assay Reagent Kit (Rockford, IL, USA). Then, an equal amount of protein (50 μg) was separated by sodium dodecyl sulfate-polyacrylamide gel electrophoresis (SDS/PAGE) and transferred to a polyvinylidene fluoride (PVDF) membrane. The PVDF membrane was incubated with homologous primary antibodies at a ratio of 1:1000 overnight at 4 °C after blocked with 5% fat-free milk. The membrane was then washed with Tris buffered saline-Tween (TBS-T), followed by incubation with HRP-conjugated goat anti-rabbit antibody in TBS-T. The target protein bands were detected using an enhanced chemiluminescence detection system (Bio-Rad, Hercules, CA, USA). GAPDH was selected as an internal reference to determine protein loading (Zhu et al. 2016).

### Fluorescent cell labelling for zebrafish embryos and drug treatments

The SW480 cells were washed with DPBS twice, then transferred to a 1.5 mL Eppendorf tube. The cells were resuspended in L-15 incomplete medium containing the cell member dye CM-Dil (5 μL/mL, V:V; Wu et al. 2018). The mixture of cells and staining dye was incubated at 37 °C for 30 min. Subsequently, the cells were washed with DPBS twice to remove the unincorporated dye the stained cells were resuspended in L-15 incomplete medium at a final concentration of 50 cells/nL.

The zebrafish xenograft model was generated by microinjection of approximately 200 cells into the yolk sac of zebrafish. After 24 h of injection, the healthy juveniles with consistent fluorescence intensity were selected for random grouping. GME at different concentrations (200, 400, and 800 μg/mL) and 5-FU (200 μM) were added to the treatment solution (zebrafish water) respectively for a drug treatment period of 48 h post fertilization (hpf). The zebrafish xenograft in GME at a drug concentration (400 μg/mL) that interfered 3 days post fertilization (dpf) and 5 dpf were photographed under a fluorescence microscope. Image J software was used to quantify the cancer cell fluorescence area and migration distance in zebrafish xenograft.

### Tumour xenografts in nude mice and drug treatments

In mice xenografts, the luciferase-expressing colon cancer cells line SW480 (SW480-luc) was used for *in vivo* bioluminescence imaging using the IVIS Lumina LT systems (PerkinElmer, CA, USA). One million SW480-luc cells were injected subcutaneously into the right flank of each mouse (day 0). After the primary tumours had reached a mean volume of about 60 mm^3^, the mice carrying tumour xenografts were arbitrarily divided into four groups (model group, 5-FU group, high-dose group 56 mg/kg/day and low-dose group 28 mg/kg/day) with six mice in each group. Drug treatment was started by feeding the mice with GME (56 and 28 mg/kg/day) orally once per day for 24 consecutive days. The 5-FU (23 mg/kg/week) was used as reference drug (positive control) and administered intraperitoneally once per week. The body weight and tumour size of the mice were monitored every 3 days (Jensen et al. 2008; Guo et al. 2017). The Tumour volume (*V*) was calculated using the formula: *V* = ½ (length × width^2^).

### Statistical analysis

All results are expressed as the mean ± standard deviation. Statistical analysis was carried out using SPSS 20.0 software (SPSS, Inc., Chicago, IL, USA). One-way analysis of variance followed by Fisher’s least significant difference *post hoc* test was used to determine the statistical differences between the control and treated samples. *p*-Value <0.05 was considered as statistically significant.

## Results

### Phytochemical analysis of GME

**Figure 1.**
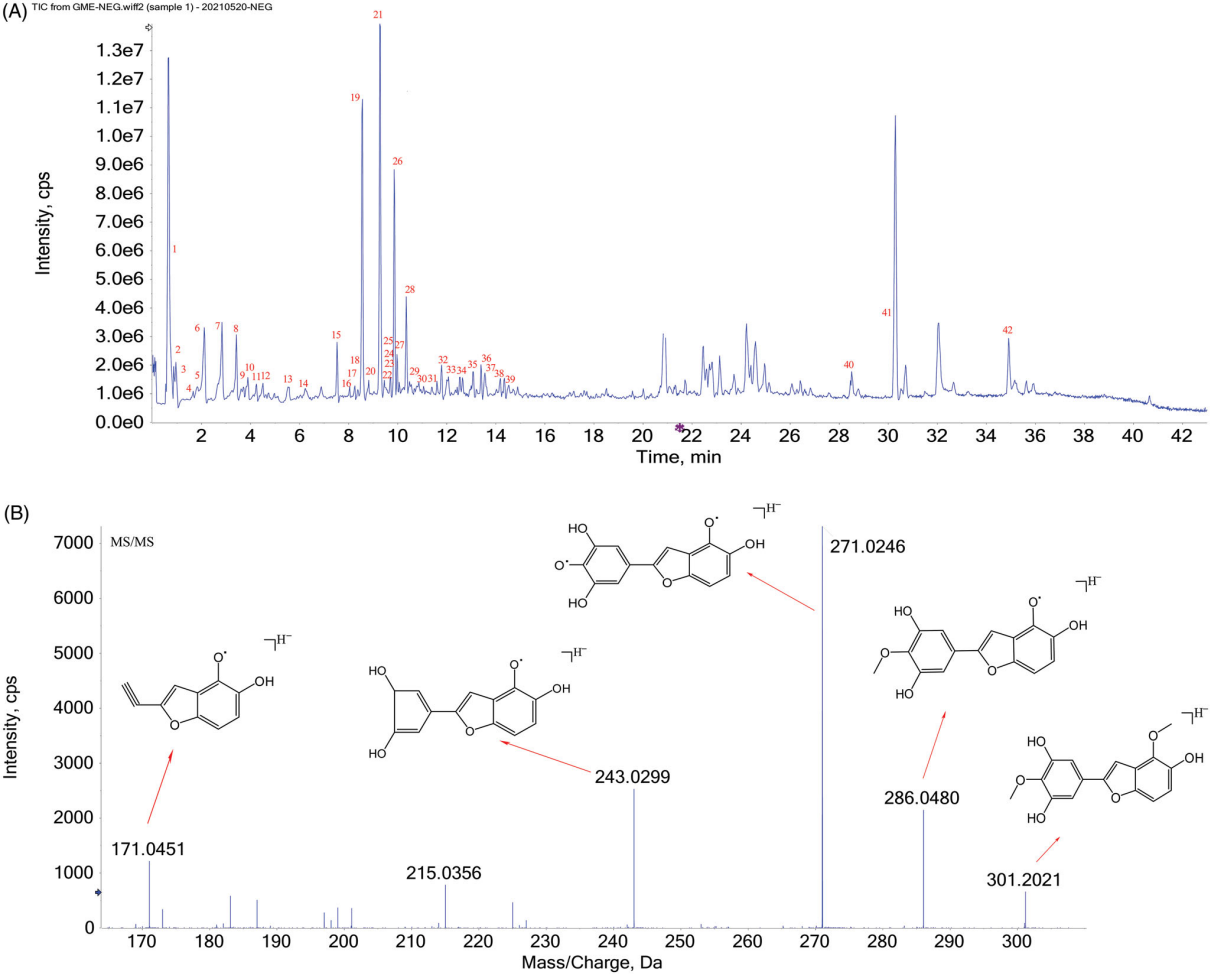
The phytochemical profile of GME using HPLC-Q-TOF-MS/MS. (A) The base peak chromatogram of GME by LC–MS in negative mode. (B) The secondary fragment ion of gnetifolin A.

The phytochemical profile of GME using HPLC-Q-TOF-MS/MS is shown in Figure 1. Figure 1(A) shows the base peak chromatogram of GME in negative ion mode and Figure 1(B) shows the secondary fragment ion of gnetifolin A. All compounds were identified through the interpretation of mass spectra obtained by the MS/MS. The identity of the compound was confirmed by aligning the mass spectra with the published data from literatures. A total of 42 compounds, including stilbenes, flavonoids, sterols, and alkaloids were identified (Table 1).

**Table 1.** Relevant analytical data of compounds isolated from GME.

No.	Retention time	Adduct/ charge	Formula	Precursor mass	Found at mass	Mass error (ppm)	Component name	MS/MS fragment ions (*m/z*)
1	0.64	[M–H]^–^	C_7_H_12_O_6_	191.0561	191.0591	–1	1-*O*-Acetyl-D-arabinofuranose	191.0591 173.0459
2	0.95	[M–H]^–^	C_8_H_11_NO_5_	200.0564	200.0566	–0.7	1-[(3-Methoxypropanoyl)oxy]-2,5-pyrrolidinedione	200.0556 182.0460 156.0663
3	1.65	[M–H]^–^	C_13_H_16_O_9_	315.0724	315.0722	0.1	Protocatechuic acid-3-glucoside	315.0722 153.0191 108.0216
4	1.82	[M–H]^–^	C_8_H_8_O_4_	167.0335	167.0348	–1.1	Vanillic acid	167.0348 152.0116 108.0217
5	1.87	[M–H]^–^	C_7_H_6_O_4_	153.0191	153.0193	–0.3	Protocatechuic acid	153.0193 108.0217
6	2.11	[M–H]^–^	C_17_H_17_NO_4_	298.1085	298.1083	0.1	*N*-(4-Acetylphenyl)-3,4-dimethoxybenzamide	298.1083 283.0851 150.0563
7	2.60	[M–H]^–^	C_16_H_17_NO_3_	270.1109	270.1135	–1.5	Dl-Demethyl-coclaurine hydrochloride	270.1135 162.0560
8	2.66	[M–H]^–^	C_7_H_6_O_3_	137.0242	137.0245	–0.9	4-Hydroxybenzoic acid	137.0245 93.0347
9	2.72	[M–H]^–^	C_17_H_19_NO_3_	284.0267	284.1292	–2.8	*N*-(4-Methylphenyl)-1-(2,3,4-trimethoxyphenyl)methanimine	284.1292 255.1026
10	3.42	[M–H]^–^	C_26_H_30_O_13_	549.1619	549.1618	0.1	Licraside	549.1618 375.1082 357.0988 327.0878
11	3.79	[M + FA–H]^–^	C_24_H_23_NO_6_	420.1454	420.1451	0.3	Unknown	466.1508 420.1454 296.0930
12	4.29	[M–H]^–^	C_32_H_46_O_16_	685.2718	685.2713	0.9	Secoisolariciresinol Diglucoside	685.2718 523.2180 418.1274
13	5.01	[M–H]^–^	C_9_H_8_O_3_	163.0541	163.0559	–2	*p*-Coumaric acid	163.0559
14	6.04	[M–H]^–^	C_14_H_12_O_4_	243.0665	243.0663	0.5	Gnetol	243.0665 225.0561 199.0761 181.2214
15	6.87	[M–H]^–^	C_21_H_24_O_9_	419.1328	419.1344	–0.9	Gnetifolin E	419.1344 257.1201 242.0582
16	7.25	[M–H]^–^	C_20_H_24_O_6_	359.1493	359.1497	–0.6	(+)-Isolariciresinol	359.1497 344.1386 313.2364 178.0631 160.0528
17	7.74	[M–H]^–^	C_26_H_32_O_11_	519.1871	519.1873	–0.2	(+)–Pinoresinol-4-*O*-beta-D-glucopyranoside	519.1873 357.1345 342.1145
18	8.02	[M–H]^–^	C_28_H_36_O_13_	579.2077	579.2081	–0.4	(–)–Syringaresinol-4-*O*-beta-D-glucopyranoside	579.2081 417.0654
19	8.36	[M–H]^–^	C_28_H_22_O_8_	485.1224	485.1233	–1.8	Gnetumontanin A	485.1182 375.0849 363.0866
20	8.53	[M–H]^–^	C_14_H_12_O_3_	227.0704	227.0718	–1.6	Resveratrol	227.0718 185.0606
21	8.62	[M–H]^–^	C_20_H_26_O_6_	361.1655	361.1658	–0.4	Secoisolariciresinol	361.1658 346.1426 315.1233 297.1204 165.0557 121.0297
22	9.30	[M–H]^–^	C_15_H_14_O_4_	257.0826	257.0823	0.3	Isorhapontigenin	257.0823 242.0558 224.0482 185.0455
23	9.32	[M–H]^–^	C_28_H_22_O_7_	469.1298	469.1294	0.4	Gnetin D	469.1293 375.1150 253.0522
24	9.68	[M–H]^–^	C_18_H_16_O_5_	311.0929	311.0924	0.5	Baicalein-5,6,7-trimethylether	311.0926 296.0685 222.8456 311.0926
25	9.71	[M–H]^–^	C_44_H_38_O_11_	741.2314	741.2326	–2	Gnetuhainin N	741.2312 617.1829 513.1548
26	9.85	[M–H]^–^	C_22_H_26_O_8_	417.1547	417.1552	–0.6	Syringaresinol	417.1175 307.0817 255.1027
27	9.96	[M–H]^–^	C_42_H_32_O_11_	711.1858	711.1872	–2.5	Gnetumontanin B	711.1875 693.1802 617.1418 587.1348
28	10.48	[M–H]^–^	C_21_H_24_O_10_	435.1303	435.1301	0.1	Isorhapontigenin-3-*O*-β-D-glucopyranoside	435.1300 255.1237 227.0714
29	10.54	[M–H]^–^	C_16_H_14_O_6_	301.2017	301.2021	–0.5	Gnetifolin A	301.2021 286.0480 271.0246 243.0299 171.0451
30	10.56	[M–H]^–^	C_15_H_12_O_5_	271.0611	271.0612	–0.1	Naringenin	271.0612 177.0459 151.1021
31	11.32	[M–H]^–^	C_36_H_36_O_13_	675.2089	675.2074	–1.3	Gnetupendin D	675.2117 513.1551 481.1335
32	11.62	[M–H]^–^	C_15_H_10_O_5_	269.0454	269.0456	–0.2	Apigenin	269.0456 225.1487 180.9889
33	11.79	[M–H]^–^	C_28_H_22_O_6_	453.1343	453.1344	–0.1	(–) *E*-Viniferin	453.1344 435.4112
34	12.07	[M–H]^–^	C_16_H_12_O_6_	299.0563	299.0562	0.1	Chrysoeriol	299.0562 284.0325 256.0375
35	13.08	[M–H]^–^	C_18_H_32_O_5_	327.2173	327.2173	0	9,12,13-Trihydroxy octadecadienoic acid	327.2173 291.1972 229.1447 211.1341 183.1393
36	13.40	[M–H]^–^	C_30_H_26_O_8_	513.1548	513.1552	–0.6	Shegansu B	513.2696 389.1027
37	13.42	[M–H]^–^	C_25_H_22_O_7_	433.1279	433.1289	–1	Gnetumontanin C	433.1289 391.1185 267.0651
38	13.78	[M–H]^–^	C_42_H_32_O_9_	679.1937	679.1959	–2.1	Gnemonol K	679.1973 585.1553
39	14.56	[M–H]^–^	C_14_H_12_O_2_	211.0756	211.0763	–0.7	Pinosylvin	211.0763 169.0661
40	28.64	[M–H]^–^	C_30_H_48_O_3_	455.3524	455.3529	–0.3	Ursolic acid	455.3513
41	30.73	[M–H]^–^	C_17_H_34_O_6_	333.2283	333.2274	–3.5	Undecyl glucoside	333.2274 275.1489 233.1030
42	34.92	[M–H]^–^	C_18_H_36_O_2_	283.2639	283.2648	–0.5	Stearic acid	283.2640

### GME inhibits the viability and morphological changes

**Figure 2.**
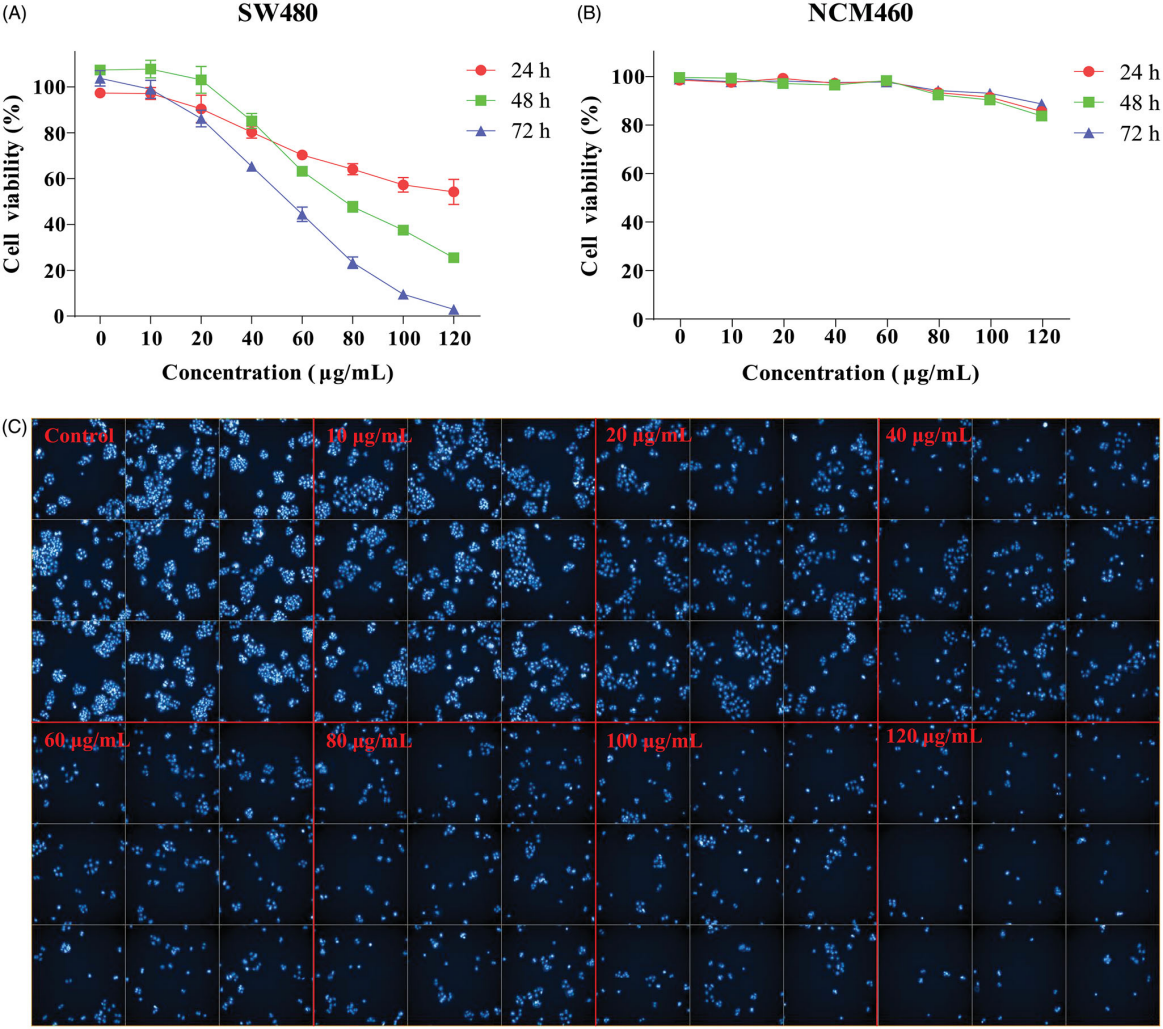
Effect of GME on the viability of colon cancer cells SW480. (A) Colon cancer cells SW480 were treated with GME at concentrations of 0–120 μg/mL in triplicates for 24, 48, and 72 h. (B) The human normal colonic epithelial cells NCM460 were treated with GME at concentrations of 0–120 μg/mL in triplicates for 24, 48, and 72 h. Cell viability was measured by MTS assay. (C) The SW480 cells were treated with GME for 48 h. Apoptotic bodies were stained with DAPI (4′,6-diamidino-2-phenylindole). The cell morphology changed significantly, and the nucleus contracted and rounded. The cells were observed under the Operetta CLS high-content analysis system at DAPI pathway.

As shown in Figure 2, GME can significantly inhibit the growth of colon cancer SW480 cells. For GME treatment at 24, 48, and 72 h, the proliferation of SW480 cells was inhibited at the half-maximal inhibitory concentration (IC_50_) of 126.50, 78.25, and 50.77 μg/mL, respectively. There was no cytotoxic effect observed on NCM460 cells at the same dose (Figure 2(B)). Figure 2 shows the effects of GME on the cell morphological changes associated with the nuclear and chromosomal condensation were observed through DAPI staining. As shown in Figure 2(C), the cell morphology was changed significantly and the nucleus was contracted and rounded when they were treated with GME for 48 h. Besides, the number of nuclei was also found gradually decreased in a dose-dependent manner.

### GME inhibits the colony-forming and migration

**Figure 3.**
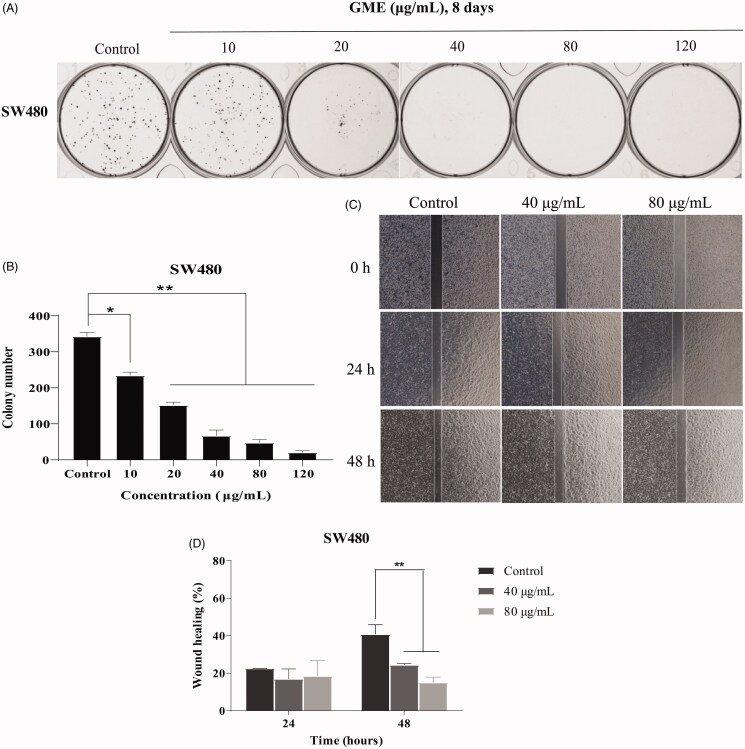
Clonogenic growth potential and migration of colon cancer cells SW480 treated with GME. (A) Evaluation of the clonogenic growth potential of SW480 cells treated with GME for 8 days. At the end of incubation, colony formation was observed by staining with crystal violet. Similar results were obtained in three independent experiments. (B) Record the colony number by a colony counter. (C) Wound healing assay was used to examine cellular migration, SW480 cells were allowed to grow into full confluence in 6-well plates, and then a wound was created with a pipette tip. GME (40 and 80 μg/mL) was added to the well and images were obtained using a microscope at 0, 24, and 48 h. Three independent experiments were examined and representative images were presented. (D) Percentage of wound healing rate. *p < 0.05; **p < 0.01.

Referring to Figure 3(A,B), the SW480 cells treated with GME for 8 days were seen to be inhibited from colony formation in a dose-dependent manner, but not in DMSO-treated controls. In addition, results of the wound healing assay that indicates the cellular migration show that the healing rates of the cell treated with GME for 24 h at concentrations of 40 and 80 μg/mL were 16.40 and 23.38%, respectively (as shown in Figure 3(C,D)). However, when the treatment time was extended to 48 h, the healing rate of the cells treated with 40 μg/mL of GME was increased to 17.56%, whereas cells treated with 80 μg/mL was reduced to 14.79%. These findings suggested that GME exhibited potent growth inhibition towards SW480 cells.

### GME induces apoptosis and cycle arrest

Figure 4(A,B) shows the increase of apoptotic cells in a dose-dependent manner. Apoptosis in the group treated with 30 μg/mL was increased from 3.06 to 9.72% when the duration of drug intervention was increased from 48 to 72 h. The same increment trend was also observed in group treated with 60 and 120 μg/mL. Apoptosis in the group of 60 μg/mL treatment was increased from 5.85 to 37.12%, whereas Apoptosis in the 120 μg/mL treatment group was increased from 20.81 to 61.53% (*p* < 0.01). Apoptosis in the 5-FU positive control group was increased 17.42–45.88% (*p* < 0.01). GME was also found to induce cell cycle arrest in colon cancer cells. Results of propidium iodide (PI) staining show that the majority of the SW480 cells were arrested in the G2/M phase (as shown in Figure 4(C,D)) after being treated with GME. The G2/M phase cells in the 30 μg/mL treatment group were increased from 20.71 to 25.95% when the duration of drug intervention was lengthened from 48 to 72 h. The G2/M phase cells in the 60 μg/mL treatment group were increased from 25.04 to 32.44%, whereas the 120 μg/mL treatment group shows an increment from 25.76 to 34.93%. The 5-FU positive control group shows an increment from 70.39 to 81.66% in the G0/G1 phase.

Figure 4.Effects of GME on apoptosis and cell cycle distribution in SW480 cells. (A) SW480 cells were treated with GME (30, 60, and 120 µg/mL) and 5-FU (20 μM) for 48 h, 72 and the cell apoptosis was detected by flow cytometry after propidium iodide (PI) and Annexin V staining. The Annexin V-negative/PI-negative cells (regarded as normal) are in the lower left quadrant and Annexin V-positive/PI-negative cells are early apoptotic cells in the lower right quadrant. Late apoptotic Annexin V-positive/PI-positive cells are in the upper right quadrant, and necrotic Annexin V-negative/PI-positive cells are in the upper left quadrant. (B) Quantitative representation of apoptotic cells in histogram. *p < 0.05; **p < 0.01. (C) Cell cycle distribution was detected by flow cytometry. (D) GME treatment for 48 h, histograms demonstrate the percentage of tumour cells at different phases of the cell cycle. (E) GME treatment for 72 h, histograms demonstrate the percentage of tumour cells at different phases of the cell cycle. Data are expressed as mean ± SD and percent of the control. **p < 0.01.

**Figure:**
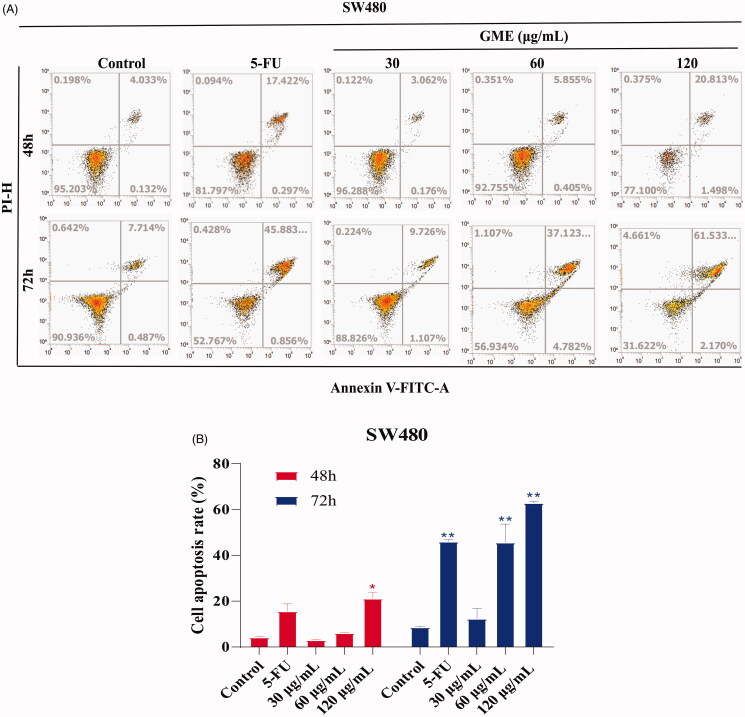

**Figure:**
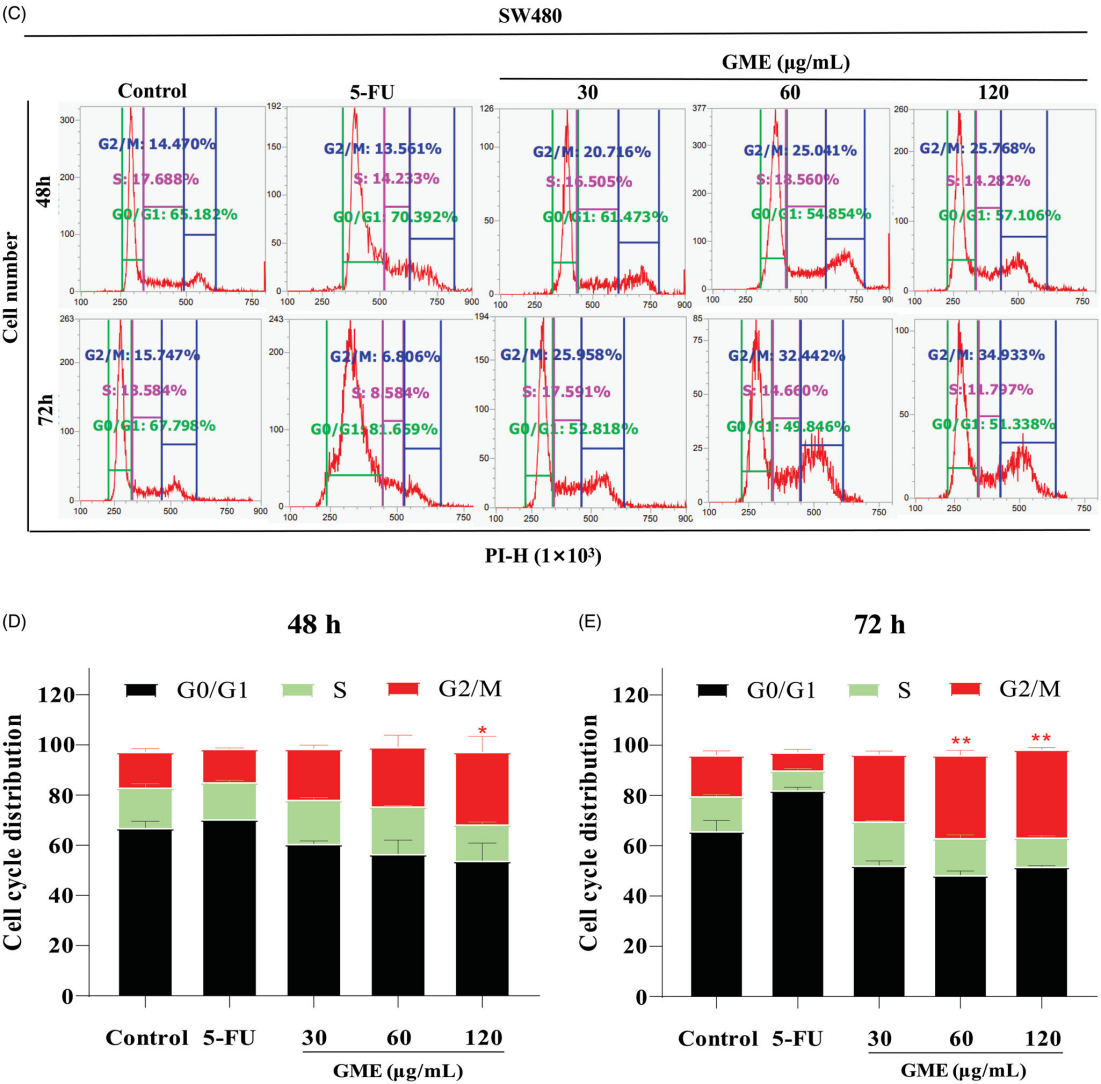

### Synergistic effect of GME and 5-FU against colon cancer

**Figure 5.**
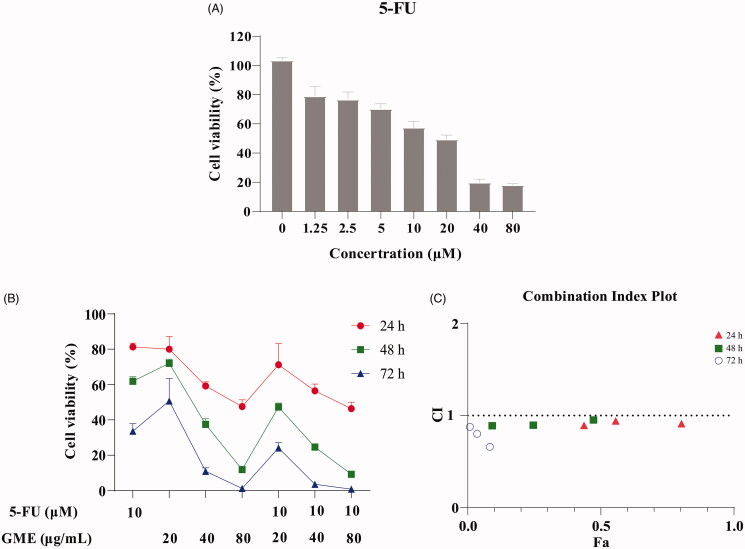
Effect of the combination of GME and 5-FU on SW480 cells. (A) The SW480 cells were treated with 5-FU (0–80 µM) for 48 h. Cell viability was measured by MTS assay. (B) The GME (20, 40, and 80 µg/mL) enhanced the anti-proliferative activity with 5-FU (10 μM), and the combination of the two has more potent synergistic effect on SW480 cells. (C) The CI values of GME and 5-FU combinations for 24, 48, and 72 h. The above data are all presented as the means ± SD of three independent experiments.

Figure 5(A) shows that 5-FU exhibited significant antitumor activity towards SW480 cells at different concentrations in the 48 h drug intervention. When combined drugs composed of 10 μM 5-FU and different concentrations of GME (20, 40, and 80 µg/mL) were used in the 24, 48, and 72 h treatment, the cell proliferation inhibitory activity was increased (Figure 5(B)). The findings reveal that the cell proliferation inhibitory effect by the combined drug of 5-FU and GME was time-dependent. The cell proliferation activity was increased with the increase of treatment time. Besides, Figure 5(C) also demonstrates that all CI values were less than 1, which indicates that there was synergistic interaction between 5-FU and GME. Among the combinations, drug composed of 10 μM 5-FU and 80 μg/mL GME displayed the most significant synergistic effect.

### Inhibitory effects of GME on AKT kinase activation

**Figure 6.**
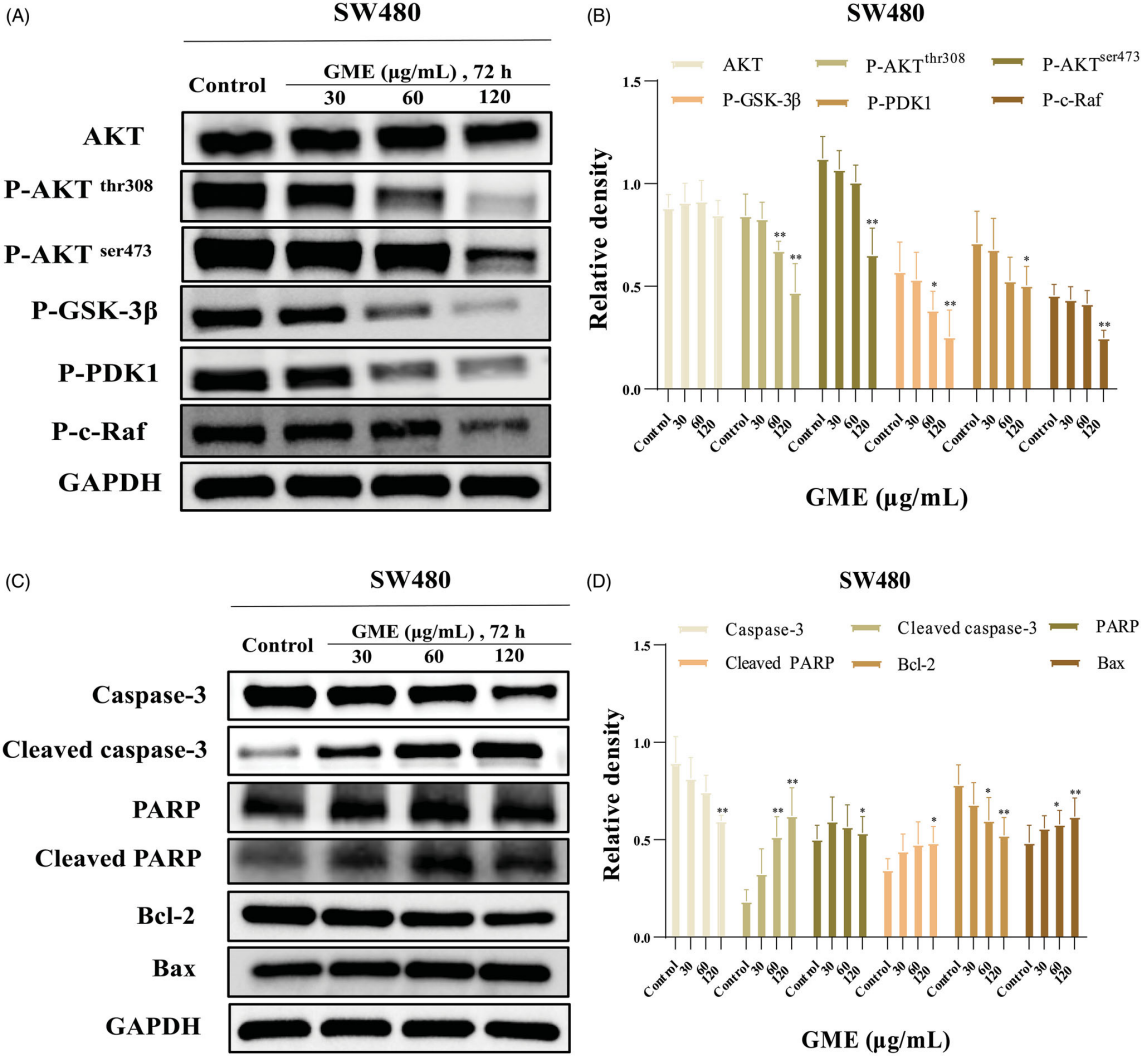
Effects of GME on PHOSPHO-AKT pathway in colon cancer cells SW480. (A) SW480 cells were treated with the absence or presence of GME for 72 h and harvested to measure protein levels of AKT, P-AKT, P-GSK-3β, P-PDK1 and P-c-Raf by western blotting. We used the housekeeping protein GAPDH as a positive loading control in all experiments. (B) Relative density of phospho-AKT signalling pathway proteins. (C) Apoptosis-related markers such as poly ADP ribose polymerase (PARP) cleavage, caspases activities and Bcl-2 family of proteins were detected by western blotting in SW480 cells. (D) Apoptosis-related protein relative density.

To verify the effect of GME on the AKT cell survival pathway, the SW480 cells were treated with different concentrations of GME for 72 h and analyzed by western blotting. Results in Figure 6(A,B) reveal that phosphorylation of AKT at Thr308 and Ser473 were decreased in a dose-dependent manner in SW480 cells. Besides, the expression of related proteins in the phospho-AKT pathway was detected and the protein expression of P-GSK-3β, P-PDK1 and P-c-Raf was also found significantly down-regulated. In addition, as shown in Figure 6(C,D), the protein expression of caspase-3 and Bcl-2 was significantly down-regulated, whereas the protein expression of cleaved caspase-3, cleaved PARP, and Bax was significantly up-regulated. These results indicate that GME induced apoptosis through the inhibition of AKT activation in the SW480 colon cancer cells.

### GME inhibits colon cancer cell migration in a zebrafish xenograft model

**Figure 7.**
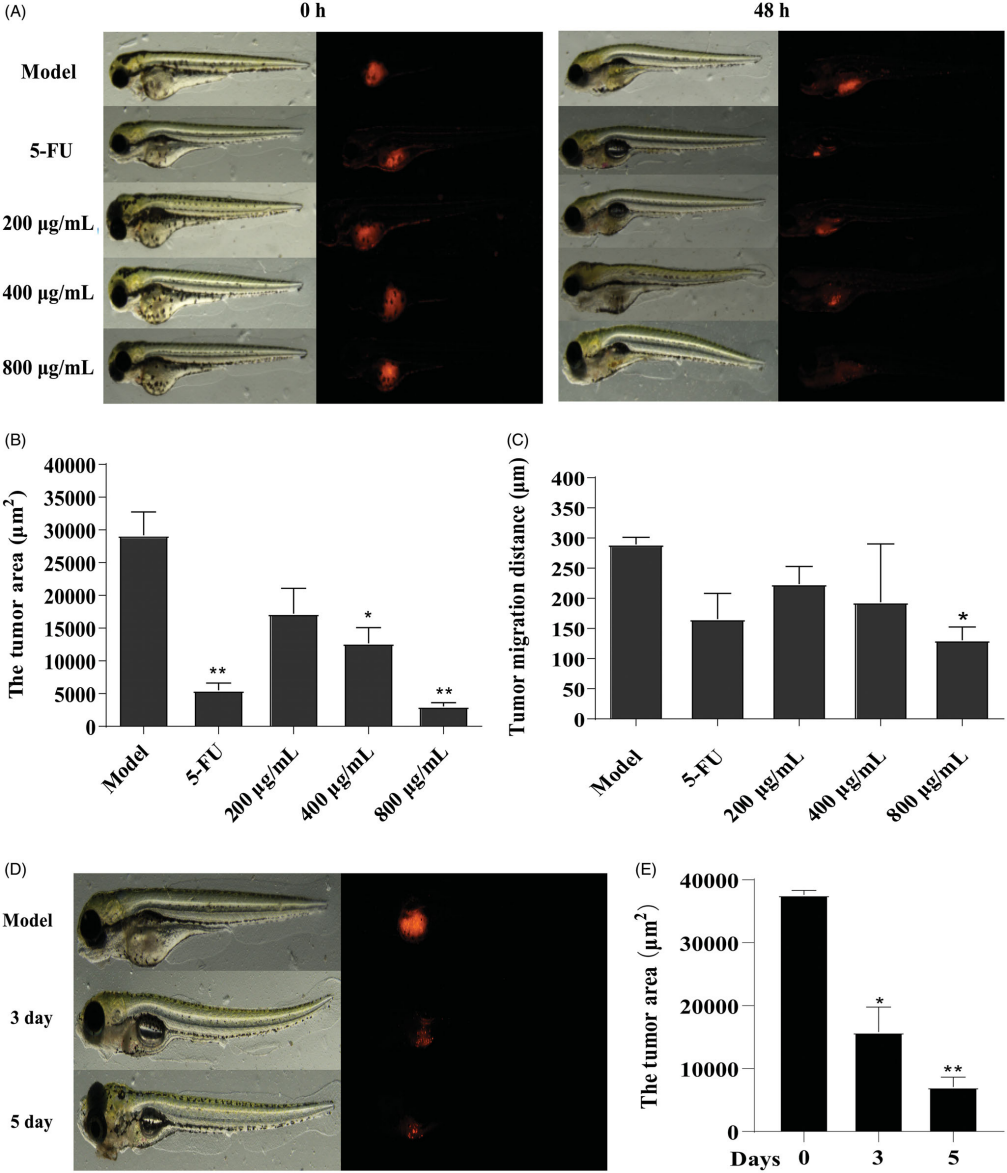
Effect of GME on xenograft zebrafish tumour model. (A) The SW480 cells were microinjected into zebrafish embryos (larvae stage, *n* = 10 per group). Fluorescence area was captured by fluorescence microscopy at 0 hpf and 48 hpf after treated with GME and 5-FU. (B) Compared with the model group, the tumour area was reduced in the treatment group. *p < 0.05; **p < 0.01. (C) Compared with the model group, the tumour migration distance was reduced in the treatment group. *p < 0.05. (D) Fluorescence area of the xenograft zebrafish tumour treated by GME (400 μg/mL) for 3 dpf and 5 dpf. (E) The tumour area significantly reduced, showing in a time-dependent manner. *p < 0.05; **p < 0.01.

A zebrafish xenograft tumour model was established to investigate the effects of GME on the proliferation and metastasis of SW480 colon cancer cells *in vivo*. In Figure 7(A), the tumour cells were seen concentrated in the yolk and the extension of the yolk in the embryo at 0 hpf under the fluorescence microscope. After treated with GME at different concentrations (200, 400, and 800 μg/mL) and 5-FU (200 μM), the area of red tumour foci (*p* < 0.01) and maximum migration distance (*p* < 0.05) of the treatment groups (as shown in Figure 7(B,C)) were significantly lower than the model group. The reduction was observed in a dose-dependent manner. Figure 7(D) shows the images of zebrafish observed under a fluorescence stereo microscope after intervened with 400 μg/mL GME for 3 and 5 dpf. The images show that the tumour foci area was significantly reduced. Figure 7(E) shows that the reduction was progressed in a time-dependent manner.

### GME inhibits growth and proliferation in nude mice xenograft model

**Figure 8.**
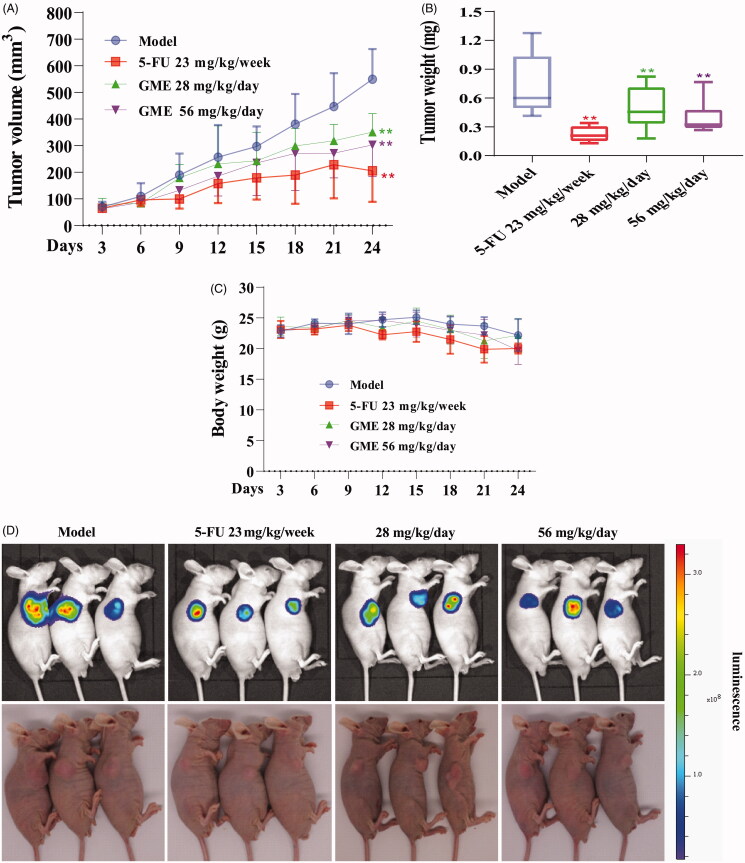
GME inhibited tumour growth in SW480-luc xenograft nude mice model. (A) Tumour volumes growth curves in treatment and model groups. Compared to model group: **p < 0.01. (B) At the end of the whole cycle, the tumour was removed and weighed. Compared with the model group, different treatment groups have significant differences: **p < 0.01. (C) Body weight measurements of mice in different groups throughout the treatment course. (D) Take white light photographs and bioluminescence imaging of SW480-luc cells xenograft mice model in different groups at the end of experiments.

To evaluate the anti-SW480 cells activity of GME *in vivo*, the human colon cancer cells SW480 was established in the nude mice xenograft model by injecting SW480-luc cells subcutaneously. Figure 8(A) shows that the tumour volumes of the treatment groups were lower than the negative control group. Figure 8(B) shows that GME reduced the tumour weight to approximately 32.19% at the dose of 28 mg/kg/day and to 53.17% at the dose of 56 mg/kg/day on day 24. In addition, there was no significant body weight lost was observed during the GME treatment, which suggesting that GME was not overtly toxic (as shown in Figure 8(C)). Besides, Figure 8(D) shows obvious difference in the bioluminescence imaging of SW480 xenograft tumours in different groups at the end of the experiments. Results of this study unveiled that GME can efficiently inhibit the growth and proliferation of SW480 cells in the nude mice xenograft model.

## Discussion

In the present study, MTS assay, colony-forming assay, migration assay, synergistic anticancer, flow cytometry analysis, western blotting, and xenograft assay were used to investigate the effects of GME on SW480 cells. Through this study, GME was proven to display significant proliferation inhibitory effect and does not exert cytotoxic effect on the NCM460 normal human colonic epithelial cells. In the LC-QTOF-MS/MS profiling analysis of GME, a total of 42 compounds were identified. Among the compounds, only resveratrol (Yuan et al. 2019), secoisolariciresinol (Bowers et al. 2019), isorhapontigenin (Subedi et al. 2019), and gnetol (Shin et al. 2018) were reported to exhibit significant antitumor effect in the previous study. Up to recently, the pharmacological action of most of the compounds in the GME is still underexplored.

The results of the MTS assay indicated that GME significantly inhibits the proliferation of SW480 cells. Besides, the results of flow cytometry analysis demonstrated that the antiproliferation activity of GME on SW480 cells was related to apoptosis and cell cycle arrest. Through DAPI staining, the nucleus was found contracted and rounded after 48 h GME treatment. This observation suggested the occurrence of apoptosis. Furthermore, the proteins P-AKT, P-GSK-3β, P-PDK1, P-c-Raf, caspase-3, and Bcl-2 were found significantly down-regulated, whereas the cleaved caspase-3, cleaved PARP, and Bax were significantly up-regulated. GME was also proved to inhibit growth and migration of SW480 cells in a dose-dependent manner. In this study, the antitumor effect of the combined drugs composed of GME and 5-FU, which is the most commonly used drug for colon cancer treatment was also investigated (Riahi-Chebbi et al. 2019; Yang et al. 2019). Combination of GME and 5-FU was found to act synergistically in inhibiting the proliferation of colon cancer cells at a lower dosage of chemotherapeutic. Furthermore, the combination also showed the potential and prospect of GME as an adjuvant drug for chemotherapy in clinical application.

AKT is an important regulator of several cellular functions such as cell growth, apoptosis, and survival (Shin et al. 2017; Zeng et al. 2019). Overexpression of AKT has been found to associate with various human malignancies. Thus, inhibiting the AKT pathway could be an effective way to prevent and treat malignancies (Debatin 2004). In the present study, the inhibition of AKT activation is potentially one of the underlying mechanisms of GME in inducing apoptosis in the SW480 human colon cancer cells. Further, GME is derived from a natural plant which possesses multi-target and multi-channel regulatory effects. This is a preliminary study to explore the regulation of AKT signalling pathway by GME. Further study to determine the mechanism of antitumor action of GME is needed for detailed pharmacodynamic study in the future.

The zebrafish xenograft tumour model has been used in numerous studies to investigate various human cancers, such as colon cancer, prostate cancer, and breast cancer (Francescangeli et al. 2015; Fior et al. 2017; Miao et al. 2021). In this study, a zebrafish xenograft tumour model was established to explore the effects of GME on the proliferation and metastasis of SW480 colon cancer cells *in vivo*. The results showed that GME significantly inhibited the proliferation and migration of SW480 cells in zebrafish. Interestingly, the similar findings were also observed in the nude mice xenograft model. This outcome suggests that the zebrafish xenograft model is superior to animal model in *in vivo* study due to its advantages of high throughput, short cycle, and convenient experiment setting, which provides an economical drug screening model for the development of antitumor drugs.

## Conclusions

The present study proved that GME exhibited significant *in vitro* and *in vivo* antitumor activity on SW480 cells. GME inhibits SW480 cell growth and migration through apoptosis induction and G2/M phase cell cycle arrest, which is realized by attenuating the AKT signalling pathway. Therefore, GME may be a novel adjuvant agent for the treatment of colon cancer.
